# Cohort population analysis of sparse data: Dexamethasone pharmacokinetics in mother and fetus based on blood sampling at birth

**DOI:** 10.1007/s10928-026-10048-5

**Published:** 2026-07-06

**Authors:** Wojciech Krzyzanski

**Affiliations:** Department of Pharmaceutical Sciences, University at Buffalo, 401 Pharmacy Building, Buffalo, NY 14214 USA

**Keywords:** Gauss-Hermite quadrature, Cohort population, Mixture population models, Dexamethasone, Parturient women, Sparse data

## Abstract

We introduced an estimation technique for mixed effect models that stems from non-parametric methods of estimation and fully parametric mixture models with cohort population as a central concept. The cohort population partitions data variability into between-cohorts and residual variabilities. This permits maximum likelihood estimates of model parameters from sparse data such as one observation per subject. Our second objective was to test robustness of cohort population estimation technique using previously published pharmacokinetic data on dexamethasone in parturient women. We performed two studies. In Study I we generated 100 dense and sparse datasets and refitted the data to quantify the difference between parameter estimates obtained by the cohort population model and the standard estimation methods. In Study II we evaluated robustness of cohort population model using dexamethasone maternal and fetal plasma concentrations obtained at childbirth after intramuscular injection of two 8 mg antenatal doses of dexamethasone phosphate in pregnant women. We implemented in NONMEM 7.6 the 3-cohort population as a mixture population model and applied first order conditional estimation, stochastic approximation, and importance sampling methods for parameter estimation. We showed that cohort estimation can quantify the variability in (log)normal populations where between-cohort variability is a substitute for between-subject variability. The cohort estimates of structural model parameters were equally accurate than the standard estimates and the accuracy and precision of estimates of inter-individual variability parameters for sparse data were better than for the standard maximum likelihood methods. Finally, we showed that the cohort population estimates provided meaningful quantification of one compartment model parameters for dexamethasone in parturient women where data were limited only to one blood sample per subject drawn after childbirth.

**Supplementary Information:**

The online version contains supplementary material available at https://doi.org/10.1007/s10928-026-10048-5.

## Introduction

Obtaining estimates of model parameters describing observations taken from subjects enrolled in a clinical study might be challenging if the number of observations per subject is less than the number of parameters. We refer to such data as sparse observations and the study design as sparse sampling. Spare sampling is common in Phase 2 and 3 clinical trials where subjects are ill patients and frequent blood sampling will impact their wellbeing. Sparse sampling designs are also selected for healthy subjects in special populations such as pregnant women, pediatric or geriatric patients. The maximum likelihood estimation of model parameters is a dominant technique in clinical data analysis that is supported by a variety of numerical algorithms implemented in pharmacometrics software. This technique estimates model parameters by maximizing the conditional probability density function of observations given model parameters (the likelihood of observations).

An example of a sparse data sampling is blood drawing from a mother during childbirth. Blood is drawn from a maternal peripheral vein and the umbilical vein at delivery. Then the data consists of two observations, one for the mother and another for the fetus. Corticosteroids are administered to pregnant women at risk of premature birth to prevent respiratory distress syndrome in newborns. Betamethasone and dexamethasone are two corticosteroids recommended for this indication. Because of corticosteroids' adverse effects on mother and fetus, knowledge of pharmacokinetics (PK) of these drugs is of great importance for dose optimization. Population PK models for antenatal corticosteroids have been developed [1].

Pharmacometric modeling approaches of sparse sampling population data include nonparametric methods, mixed effects models, and Bayesian estimations. Nonparametric methods do not assume a particular shape of the parameter distribution but rather describe it by a finite number of point distributions (centered at support points) with associated probabilities. By contrast, parametric methods assume that parameter distribution can be described by a vector of parameters (typically multivariate normally distributed). Parameter distributions are more susceptible to data sparseness than nonparametric distributions [2]. Although our focus is on parametric methods, the nonparametric approach is a viable alternative for analysis of sparse clinical data [3]. Nonlinear mixed effect models are primary tools for handling clinical data analysis. They are parametric techniques that maximize the likelihood function of observations to obtain estimates of model parameters. Sparse data creates a challenge as most numerical algorithms maximizing the likelihood require individual parameter estimates that tend to be close to the population means when individual observations are insufficient. The estimation methods based on explicit but approximate description of the likelihood function (such as first order conditional estimation, FOCE) are inferior to exact expectation maximization (EM) methods (such as importance sampling, IMP, and stochastic approximation, SAEM) when handling sparse data [4]. EM methods are used in analysis of sparse pharmacokinetic data in pediatric populations [5]. In Bayesian analysis, the joint posterior distribution of unobserved stochastic quantities (including model parameters) conditional on the observed data is calculated from the distribution of observed data conditional on unobserved stochastic quantities and their prior distribution. The model parameters are inferred from the joint posterior distribution rather than from maximizing the likelihood of observations. The pros and cons of Bayesian vs. nonlinear mixed effects approaches in PK and pharmacodynamic (PD) data analysis are discussed elsewhere [6]. The Bayesian estimation of individual PK parameters is applied in therapeutic drug monitoring of critically ill patients where sparse data are inherent [7].

A numerical challenge for maximum likelihood estimation in nonlinear mixed effects models is the evaluation of the likelihood function, because it involves the evaluation of a multiple integral that, in general, does not have an explicit solution. The Gauss quadrature approximation of the likelihood function utilizes the Gauss-Hermite quadrature rules of integration [8]. The quadrature rules specify the nodes and weights such that the integral is approximated by the weighted sum of the integrand evaluated at the nodes. From the nonparametric point of view, the nodes can be interpreted as the support points for point distributions and the weights as the probabilities. Then the weighted sum of point distributions becomes a discrete likelihood function that approximates the continuous likelihood of observations. This interpretation of the Gauss quadrature approximation serves as a basis for an estimation technique introduced in this report.

The objective of this work is to introduce a technique of parameter estimation from sparse population data that stems from the nonparametric description of the likelihood function but is fully parametric. Our work is divided into two studies: proof of concept where the technique is introduced and compared to FOCE, IMP, and SAEM estimation methods for simulated data, and a case study where we apply this technique to estimate PK parameters for dexamethasone measured in mother and fetus during birth when there is only one observation available.

## Thoretical

### Gauss-Hermite cohort population

Let * X* be a random variable defined on a population of subjects that assumes a finite number of values $\:{x}_{1},$ …, $\:{x}_{N}$. We call this population a cohort population, where the * i*th cohort is the set of subjects for which $\:X={x}_{i}$. * X* can be considered a continuous random variable with the probability density function equal to the sum of point distributions centered on at $\:{x}_{1},$ …, $\:{x}_{N}$:

1 $$\:{p}_{X}\left(x\right)={\sum\:}_{i=1}^{N}{w}_{i}\delta\:(x-{x}_{i})$$

where $\:\delta\:\left(x-{x}_{i}\right)$ is the Dirac delta function centered at $\:{x}_{i}$, and $\:{w}_{1},$ …, $\:{w}_{N}>0$ are positive numbers such that

2 $$\:{w}_{1}+\:\dots\:\:{+\:w}_{N}=1$$

$\:{w}_{i}$ is the probability of finding a subject in the population that belongs to the * i*th cohort. The * k*th moment of the cohort distribution is equal to

3 $${m}_{k}={\int\:}_{-\infty\:}^{\infty\:}{x}^{k}{p}_{X}\left(x\right)dx={\sum\:}_{i=1}^{N}{w}_{i}{x}_{i}^{k}$$

In particular, the mean of * X*:

4 $$\:E\left(X\right)={m}_{1}=\:{\sum\:}_{i=1}^{N}{w}_{i}{x}_{i}$$

and the variance of * X*:

5 $$\:Var\left(X\right)={m}_{2}-{m}_{1}^{2}=\:{\sum\:}_{i=1}^{N}{w}_{i}{x}_{i}^{2}-{\left({\sum\:}_{i=1}^{N}{w}_{i}{x}_{i}\right)}^{2}$$

The Gauss-Hermite quadrature rule states that for a smooth function $\:f\left(x\right)$ and a positive integer * N* there exists a number $\:{\xi\:}^{\left(N\right)}$ such that [9]

6 $$\:{\int\:}_{-\infty\:}^{\infty\:}f\left(x\right){e}^{-{x}^{2}}dx={\sum\:}_{i=1}^{N}{w}_{i}^{\left(N\right)}f\left({\xi\:}_{i}^{\left(N\right)}\right)+\frac{N!\sqrt{\pi\:}}{{2}^{N}\left(2N\right)!}\frac{{d}^{2N}f}{{dx}^{2N}}\left({\xi\:}^{\left(N\right)}\right)$$

where $\:{\xi\:}_{1}^{\left(N\right)}<\dots\:<{\xi\:}_{N}^{\left(N\right)}$ are roots of the Hermite polynomial of degree * N* $\:{H}_{N}\left(x\right)$ (called the quadrature nodes) and

7 $$\:{w}_{i}^{\left(N\right)}=\:{2}^{N+1}N!\sqrt{\pi\:}/{\left[{H}_{N+1}\left({\xi\:}_{i}^{\left(N\right)}\right)\right]}^{2}$$

(called the quadrature weights). Table S1 lists nodes and weights for Gauss-Hermite quadrature rules up to $\:N\le\:5$ [10]. Equation (6) implies that if $\:f\left(x\right)$ is a polynomial of degree less than * 2N*, the remainder vanishes, and the integral is equal to the sum. If $\:f\left(x\right)$ is an arbitrary smooth function the sum is used as an approximation of the integral and the remainder is the error of approximation. Also, one can calculate that

8 $$\left.\begin{array}{l} \:{\int\:}_{-\infty\:}^{\infty\:}f\left(x\right)\frac{1}{\sqrt{2\pi\:{\omega\:}^{2}}}{e}^{-({x-\theta\:)}^{2}/\left(2{\omega\:}^{2}\right)} dx\\ \approx\:{\sum\:}_{i=1}^{N}\frac{{w}_{i}^{\left(N\right)}}{\sqrt{\pi\:}}f\left(\theta\:+\sqrt{2}\omega\:{\xi\:}_{i}^{\left(N\right)}\right) \end{array}\right.$$

and Eq. (8) is exact if $\:f\left(x\right)$ is a polynomial of degree less than * 2N.* For $\:f\left(x\right)\equiv\:1$, Eq. (8) becomes

9 $$\:1={\sum\:}_{i=1}^{N}\frac{{w}_{i}^{\left(N\right)}}{\sqrt{\pi\:}}$$

This property of the Gauss-Hermite quadrature defines the Gauss-Hermite N-cohort population given by the following p.d.f.:

10 $$\:{p}_{N}\left(x\right)={\sum\:}_{i=1}^{N}\frac{{w}_{i}^{\left(N\right)}}{\sqrt{\pi\:}}\delta\:\left(x-\theta\:-\sqrt{2}\omega\:{\xi\:}_{i}^{\left(N\right)}\right)$$

Equation (8) implies that

11 $$\left.\begin{array}{l}\:{\int\:}_{-\infty\:}^{\infty\:}f\left(x\right)\frac{1}{\sqrt{2\pi\:{\omega\:}^{2}}}{e}^{-({x-\theta\:)}^{2}/\left(2{\omega\:}^{2}\right)} dy\\ \approx\:{\int\:}_{-\infty\:}^{\infty\:}f\left(x\right){p}_{N}\left(x\right)dx \end{array}\right.$$

**Fig. 1:**
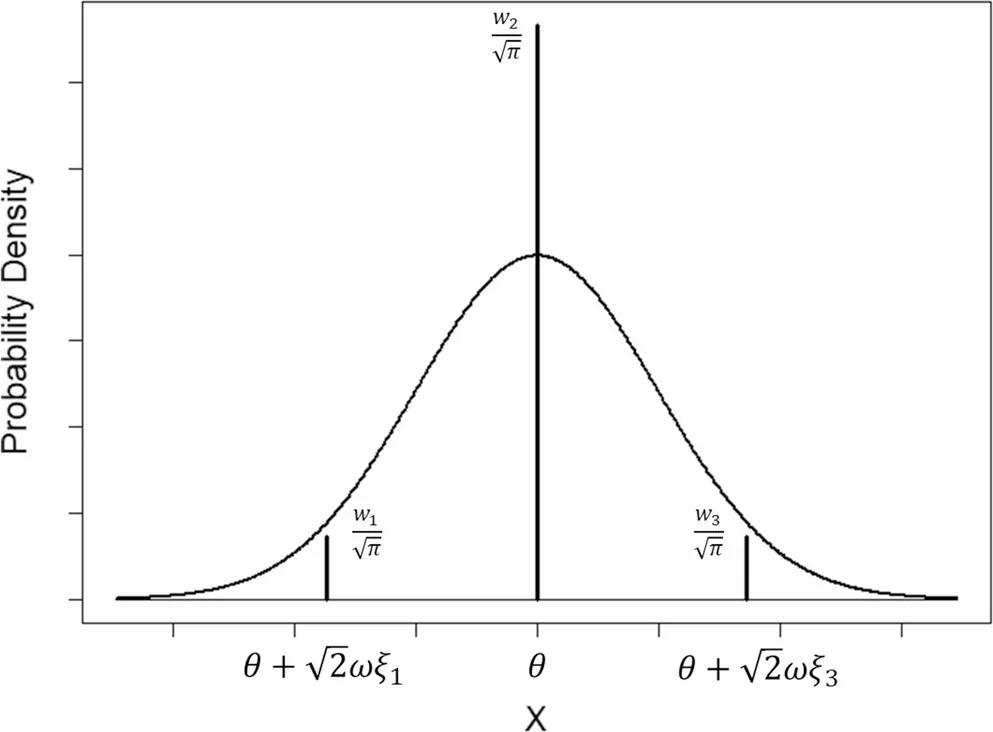
Approximation of the probability density function for normal distribution with the mean $\:\theta\:=0\:$ and standard deviation $\:\omega\:=1\:$ with the 3-cohort Gauss-Hermite p.d.f

Equation (11) states that $\:{p}_{N}\left(x\right)$ approximates the normal distribution in the distributional sense as * N* becomes large. Moreover, Eq. (11) becomes exact for $\:f\left(x\right)={x}^{k}$, $\:k\le\:2N-1$. This means $\:{p}_{N}\left(x\right)$ has the same moments up to * 2N-1* as the normal distribution. In particular, the mean and standard deviation of Gauss-Hermite N-cohort population are $\:\theta\:$ and $\:\omega\:$, respectively, given $\:N\ge\:2$. The graphs of $\:{p}_{N}\left(x\right)$ for * N=3* overlaid with the graph of the normal p.d.f. with the same $\:\theta\:$ and $\:\omega\:$ are shown in Fig. 1.

### Gauss-Hermite cohort population as a mixture population

A mixture population arises if a population consists of finite number of subpopulations with different characteristics. The distribution of a random variable defined on a mixture population will exhibit multimodal shape with each mode contributing to a single mode subpopulation. Mixture population models typically involve the p.d.f.s that are a barycentric combination of normal or log-normal distributions where the barycentric coordinates are called mixing parameters interpreted as probabilities of finding a subject in particular subpopulation. Letting the variances in such unimodal distributions to zero results in a mixture population of point distributions, that we call cohorts. Using this terminology, the Gauss-Hermite N-cohort population can be considered as a mixture population of N cohorts. The mixture population models are common in pharmacometrics and many pharmacometric programs support their applications. Consequently, the Gauss-Hermite cohort population models can be implemented in pharmacometric software as mixture population models with variances set to zero (or being close to zero), the mixing parameters equal to $\:{w}_{i}^{\left(N\right)}/\sqrt{\pi\:}$, and the means equal to $\:\theta\:+\sqrt{2}\omega\:{\xi\:}_{i}^{\left(N\right)}$.

## Methods

We performed two studies. In Study I we generated dense and sparse datasets and refitted the data to demonstrate that the Gause-Hermite cohort population model can accurately estimate model parameters whereas the standard estimation methods cannot. In Study II we used sparse clinical data to evaluate the robustness of Gauss-Hermite cohort population model.

### Study I: Proof of concept

#### Data

**Fig. 2:**
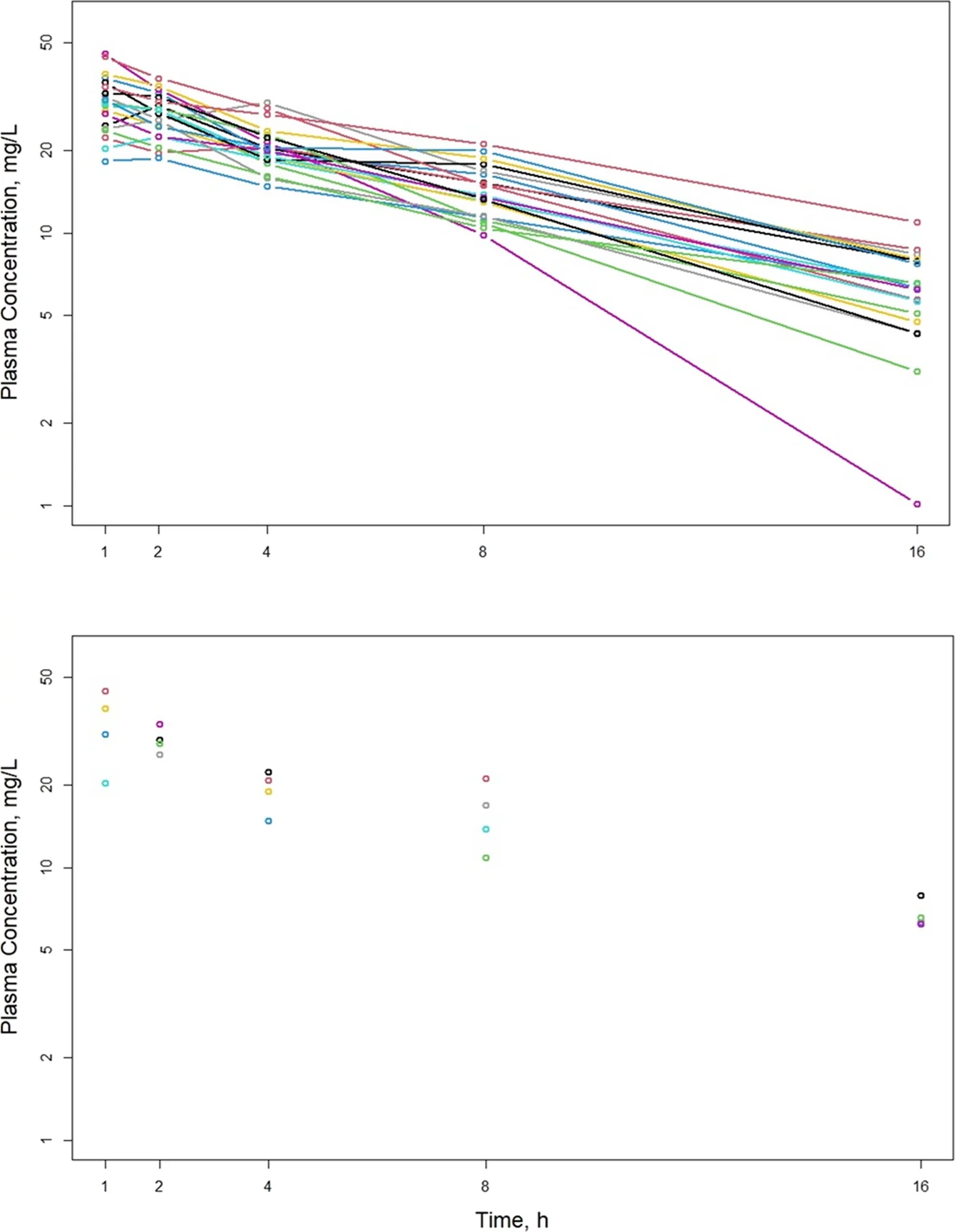
Upper panel: Simulated time courses of drug plasma drug concentrations for 20 subjects using one compartmental model with IV bolus input of dose 100 mg. Each subject had 5 observations at times 1, 2, 4, 8, and 16 h. Lower panel: Drug plasma concentrations randomly selected for 20 subjects from the dense dataset such that each subject had only 1 observation and there were 4 observations at each time point. Parameter values used for simulations are shown in Table 1. For Study I 100 datasets with 20 subjects were simulated

One compartment model with an intravenous bolus dose of 100 mg was used to simulate 100 sets of drug plasma concentrations in 20 subjects at times 1, 2, 4, 8 and 16 h. The * CL* and * V* values were log-normally distributed with the means 0.3 L/h and 3 L, and variances 0.04 (log(L/h))^2^, and 0.09 (log(L))^2^, respectively. The proportional residual error was applied with the coefficient of variation * CV=0.1*. Simulations were performed using RStudio 2026.01.2 (Posit Software PPC). The sparse dataset consisted of one observation per subject such that each observation time was shared by 4 subjects. The plots of dense and sparse plasma concentrations for one of 100 data sets are shown on Fig. 2.

#### Data analysis

Both dense and sparse datasets were analyzed using one-compartment model where the inter-individual variability (IIV) of log-normally distributed * CL* and * V* was modeled by 1-cohort population model

12 $$\log (\:CL)\sim\delta\:\left(x-{\mathrm{l}\mathrm{o}\mathrm{g}\theta\:}_{CL}\right)$$

13 $$\log\:(V)\sim\delta\:\left(x-{\mathrm{l}\mathrm{o}\mathrm{g}\theta\:}_{V}\right)$$

and N×N-cohort population model, *N* = 2, 3, 4, 5:

14 $$\log\:(CL)\sim{\sum\:}_{i=1}^{N}\frac{{w}_{i}^{\left(N\right)}}{\sqrt{\pi\:}}\delta\:\left(x-{\mathrm{l}\mathrm{o}\mathrm{g}\theta\:}_{CL}-\sqrt{2}{\omega\:}_{CL}{\xi\:}_{i}^{\left(N\right)}\right)$$

15 $$\log\:(V)\sim{\sum\:}_{i=1}^{N}\frac{{w}_{i}^{\left(N\right)}}{\sqrt{\pi\:}}\delta\:\left(x-{\mathrm{l}\mathrm{o}\mathrm{g}\theta\:}_{V}-\sqrt{2}{\omega\:}_{V}{\xi\:}_{i}^{\left(N\right)}\right)$$

and binormal covariance matrix

16 $$\log\:(CL)\sim\mathcal{N}({\mathrm{l}\mathrm{o}\mathrm{g}\theta\:}_{CL},{{\omega\:}_{CL}}^{2})$$

17 $$\log\:(V)\sim\mathcal{N}({\mathrm{l}\mathrm{o}\mathrm{g}\theta\:}_{V},{{\omega\:}_{V}}^{2})$$

The proportional residual error model used for generating the data was used for parameter estimation:

$$\left.\begin{array}{l}{C}_{ij}=\mathrm{C}\left({t}_{ij}\right)+{\mathrm{C}\left({t}_{ij}\right)\epsilon\:}_{ij}\:,\\ \:{\epsilon\:}_{ij}\:\sim\mathcal{N}(0,{CV}^{2})\end{array}\right.$$

where $\:{C}_{ij}$ is the observed drug plasma concentration for subject * i* at time $\:{t}_{ij}$, $\:\mathrm{C}\left({t}_{ij}\right)$ is the model predicted drug plasma concentration, and * CV* is the coefficient of variation for the residual error. Population analysis was performed using NONMEM 7.6 (ICON Plc). The 1-cohort population model was implemented as naïve pooled data. The N×N-cohhort model was coded using mixture of N^2^ populations representing the product of N×N cohorts of the means:

19 $$\:\left({\mathrm{l}\mathrm{o}\mathrm{g}\theta\:}_{CL}+\sqrt{2}{\omega\:}_{CL}{\xi\:}_{i}^{\left(k\right)}\right)\left({\mathrm{l}\mathrm{o}\mathrm{g}\theta\:}_{V}+\sqrt{2}{\omega\:}_{V}{\xi\:}_{j}^{\left(N\right)}\right),\:i,j=1,\dots\:,\:N$$

and 0 variances with mixing parameters fixed at $\:{w}_{i}^{\left(N\right)}{w}_{j}^{\left(N\right)}/\pi\:$, $\:i,j=1,\dots\:,\:N$. The first order conditional estimation (FOCE) method was used for parameter estimation. Additionally, for the IIV model Eqs. (16)-(17) the IMP and SAEM estimation methods were applied as they were reported to preform better than FOCE for parameter estimation from sparse data [4, 11]. The convergence criteria for IMP and SAEM methods were of CTYPE=3 that mimicked the convergence criteria for FOCE. The maximum number of iterations was set ITER=500 and the default sample size was used. All estimation methods required 3 digits of accuracy.

#### Assessment of model performance

All models were assessed based on success rate of estimation method, and accuracy and precision of estimates of model parameters obtained for dense and sparse datasets. The success rate of an estimation method was the percent of estimations out of 100 datasets that resulted in the non-singular covariance matrix for parameter estimates. The accuracy could be quantified since the true parameter values used for generating the data were known. The parameter estimate was represented by the arithmetic mean of the successful estimates out of 100 datasets. The accuracy was quantified by the relative difference between the mean and True Value:

20 $$\:R\varDelta\:P=\frac{100\%}{\mathrm{T}\mathrm{r}\mathrm{u}\mathrm{e}\:\mathrm{V}\mathrm{a}\mathrm{l}\mathrm{u}\mathrm{e}}\frac{1}{{N}_{succ}}\sum\:_{i=1}^{{N}_{succ}}\left({\mathrm{E}\mathrm{s}\mathrm{t}\mathrm{i}\mathrm{m}\mathrm{a}\mathrm{t}\mathrm{e}}_{\mathrm{i}}-\mathrm{T}\mathrm{r}\mathrm{u}\mathrm{e}\:\mathrm{V}\mathrm{a}\mathrm{l}\mathrm{u}\mathrm{e}\right)$$

where $\:{N}_{succ}$ is the number of successful estimations, Estimate_i_ is the parameter estimate for the i-th data set, i = 1,…, $\:{N}_{succ}$, and True Value is the parameter value used for simulations. The precision was assessed by the relative root mean square error (%RRMSE):

21 $$\:\%RRMSE=\frac{100\%}{\mathrm{T}\mathrm{r}\mathrm{u}\mathrm{e}\:\mathrm{V}\mathrm{a}\mathrm{l}\mathrm{u}\mathrm{e}}\sqrt{\frac{1}{{N}_{succ}}\sum\:_{i=1}^{{N}_{succ}}{\left({\mathrm{E}\mathrm{s}\mathrm{t}\mathrm{i}\mathrm{m}\mathrm{a}\mathrm{t}\mathrm{e}}_{\mathrm{i}}-\mathrm{T}\mathrm{r}\mathrm{u}\mathrm{e}\:\mathrm{V}\mathrm{a}\mathrm{l}\mathrm{u}\mathrm{e}\right)}^{2}}$$

### Study II: Dexamethasone pharmacokinetics in parturient women

#### Data

The 1-cohort and 3-cohort population models were applied to describe the time courses of dexamethasone (DEX) in parturient women and their fetuses based on a single blood drawing from the peripheral and umbilical cord veins at birth [12]. Fourteen near term women to deliver by cesarian section were given intramuscularly a dose 8 mg of dexamethasone phosphate (equivalent to 6.67 mg of DEX) at noon a day prior to delivery and a second dose 8 mg of dexamethasone phosphate at 10 am on the day of delivery. Plasma samples were stored at -80 °C until analyzed for DEX concentrations using high performance liquid chromatography. Maternal and fetal DEX plasma concentrations and times after the last dose of each subject are shown in Table S2. The time after the first dose was calculated by adding 22 h to the time after the last dose.

#### Pharmacokinetic model

We applied a one-compartment model with first-order input from the intramuscular compartment and first-order elimination to describe maternal plasma concentrations $\:{C}_{m}$:

22 $$\:\frac{d{A}_{IM}}{dt}=FDose\delta\:\left(t\right)-FDose\delta\:\left(t-{t}_{2}\right)-{k}_{a}{A}_{IM},\:{A}_{IM}\left(0\right)=0$$

23 $$\:\frac{d{A}_{p}}{dt}={k}_{a}{A}_{IM}-\frac{CL}{V}{A}_{p},\:{A}_{p}\left(0\right)=0$$

24 $$\:{C}_{m}=\frac{{A}_{p}}{V}$$

where $\:{A}_{IM}\left(t\right)$ is DEX amount in the intramuscular compartment at time * t* after first $\:Dose=\:$8000 µg, $\:{A}_{p}\left(t\right)$ is the amount of DEX in the plasma compartment, * F* is the bioavailability, $\:{k}_{a}$ is the first-order absorption rate constant, * CL* is the DEX clearance, * V* is the volume of distribution, and $\:{t}_{2}=22\:$h is the time of second dose administration. The DEX plasma concentration in fetus $\:{C}_{f}$ was assumed to be proportional to $\:{C}_{m}$:

25 $$\:{C}_{f}=FMR\cdot\:{C}_{m}$$

where * FMR* denotes the fetal-to-maternal DEX concentration ratio.

#### Population model

The naive pooled data (1-cohort) model and 3-cohort population model were applied. The Gauss-Hermite distribution of $\:\mathrm{l}\mathrm{o}\mathrm{g}(CL/F)$ was assumed:

26 $$\log\:(CL/F)\sim{\sum\:}_{i=1}^{3}\frac{{w}_{i}^{\left(3\right)}}{\sqrt{\pi\:}}\delta\:\left(x-{\mathrm{l}\mathrm{o}\mathrm{g}\theta\:}_{CL}-\sqrt{2}\omega\:{\xi\:}_{i}^{\left(3\right)}\right)$$

For the remaining model parameters 1-cohort Gauss-Hermite distributions were assumed (fixed effect parameters):

27 $$\:V/F\sim\delta\:\left(x-{\theta\:}_{V}\right),\:{k}_{a}\sim\delta\:\left(x-{\theta\:}_{ka}\right),\:FMR\sim\delta\:\left(x-{\theta\:}_{FMR}\right)$$

The constant residual error model for log-transformed observed DEX plasma concentrations was used:

28 $$\:{\mathrm{l}\mathrm{o}\mathrm{g}(C}_{mi})={\mathrm{l}\mathrm{o}\mathrm{g}(C}_{m}\left({t}_{i}\right))+{\epsilon\:}_{mi},\:{\epsilon\:}_{mi}\:\sim\mathcal{N}(0,{{\sigma\:}_{m}}^{2})$$

29 $$\:{\mathrm{l}\mathrm{o}\mathrm{g}(C}_{fi})={\mathrm{l}\mathrm{o}\mathrm{g}(C}_{f}\left({t}_{i}\right))+{\epsilon\:}_{fi},\:{\epsilon\:}_{fi}\:\sim\mathcal{N}(0,{{\sigma\:}_{f}}^{2})$$

Since measurements of DEX concentrations were obtained from two tissues (maternal peripheral vein and umbilical cord vein), we allowed $\:{{\sigma\:}_{m}}^{2}$ and $\:{{\sigma\:}_{f}}^{2}$ to differ.

#### Data analysis

The population models were implemented in NONMEM 7.6. The differential equations Eqs. (22)-(23) were solved:

30 $$\left.\begin{array}{l}C_m(t)=\begin{cases}\frac{Dose}{V\;/\;F}\frac{k_a}{k_a-CL/V}\Big(exp(-\;\frac{CL}Vt)-exp(-k_at)\Big), ~~~ 0< t \leq t_2\\C_m(t_2)+\;\frac{Dose}{V/F}\frac{k_a}{k_a-CL/V}\Big(exp(-\frac{CL}V(t-t_2))-exp(-k_a(t-t_2))\Big),~~~t>t_2\end{cases} \end{array}\right.$$

For the 1-cohort model IIV of $$\:\mathrm{l}\mathrm{o}\mathrm{g}(CL/F)$$ were fixed at 0. For the 3-cohort population model, the mixture population was applied with 3 subpopulations. The mixing parameters were set to $$\:{w}_{i}^{\left(3\right)}/\sqrt{\pi\:}$$, $$\:i=1,\:2,\:3.$$ The IIVs for $$\:\mathrm{l}\mathrm{o}\mathrm{g}(CL/F)$$ were set to 0 and the typical values were equal $$\:{\mathrm{l}\mathrm{o}\mathrm{g}\theta\:}_{CL}+\sqrt{2}\omega\:{\xi\:}_{i}^{\left(3\right)}$$, $$\:i=1,\:2,\:3,\:{\mathrm{w}\mathrm{h}\mathrm{e}\mathrm{r}\mathrm{e}\:\theta\:}_{CL}$$ and $$\:\omega\:$$ were fixed effect parameters. The first order conditional estimation (FOCE) method was used for parameter estimation. The individual * CL/F* were assigned based on the highest probability of finding a subject in one of 3 cohorts. Such probabilities can be reported in a NONMEM $TABLE record by the BESTSUB = MAXEST statement. The NONMEM control stream for the 3-cohort population model is provided in Appendix 1.

#### Assessment of model performance

The model performance was evaluated using the objective function value, relative standard errors of parameter estimates (%RSE) calculated as the ratio of the estimate by its standard error. The diagnostic plots that included observed and model predicted vs. time, observed vs. predicted, weighted residuals vs. time, and visual predictive check (VPC). To generate VPC plots, 1000 data sets were simulated at observed times for individual subjects sampled from the cohort populations. The diagnostic plots were obtained using RStudio 2026.01.2. The vpc package was applied to generate the VPC plots.

## Results

### Study I

In this study we compared estimates of one-compartment model parameters obtained by fitting dense and sparse datasets for nine estimation methods. The success rates, estimates and %RRMSEs are shown in Tables 1 and 2. To facilitate the comparisons with the true values we also calculated the relative differences of the mean estimates and %RRMSEs for dense and sparse datasets which are shown in Tables S3 and S4.

**Table 1:** The estimates of model parameters obtained by fitting 100 datasets of *N* = 20 subjects with DENSE observations. The results are presented as the means (%RRSM) of the successful estimations

Estimation Method	S.*R*. %	$\:{\theta\:}_{CL}$ L/h	$\:{\theta\:}_{V}$ L	$\:{\omega\:}_{CL}$ log(L)	$\:{\omega\:}_{V}$, log(L/h)	$\:{\omega\:}_{CL}^{2}$ log^2^(L/h)	$\:{\omega\:}_{V}^{2}$ log^2^(L)	* CV* ^2^
True Value	NA	0.3	3.0	0.2	0.3	0.04	0.09	0.01
1-cohort	100	0.305 (4.4)	3.01 (6.4)	NA	NA	NA	NA	0.0734 (656)
2 × 2-cohort	100	0.310 (11.1)	2.95 (12.1)	0.165 (39.3)	0.240 (39.7)	NA	NA	0.030 (209)
3 × 3-cohort	100	0.306 (6.9)	2.97 (10.6)	0.160 (43.2)	0.250 (28.5)	NA	NA	0.0181 (88.0)
4 × 4-cohort	100	0.308 (8.3)	2.98 (11.1)	0.196 (17.1)	0.289 (16.2)	NA	NA	0.0154 (59.9)
5 × 5-cohort	100	0.308 (7.2)	2.94 (10.2)	0.182 (18.2)	0.261 (19.1)	NA	NA	0.0136 (43.0)
FOCE	100	0.305 (4.4)	3.10 (6.9)	NA	NA	0.0376 (34.9)	0.0845 (34.5)	0.0106 (20.2)
IMP	100	0.303 (4.2)	3.01 (6.9)	NA	NA	0.0376 (34.9)	0.0767 (34.5)	0.0103 (19.8)
SAEM	100	0.302 (4.2)	3.00 (6.9)	NA	NA	0.0376 (34.9)	0.0846 (34.5)	0.0103 (18.6)

*S.R.* Success rate

*NA* Not available

**Table 2:** The estimates of model parameters obtained by fitting 100 datasets of *N* = 20 subjects with SPARSE observations. The results are presented as the means (%RRSME) of the successful estimations

Estimation Method	S.*R*. %	$\:{\theta\:}_{CL}$ L/h	$\:{\theta\:}_{V}$ L	$\:{\omega\:}_{CL}$ log(L/h)	$\:{\omega\:}_{V}$, log(L)	$\:{\omega\:}_{CL}^{2}$ log^2^(L/h)	$\:{\omega\:}_{V}^{2}$ log^2^(L)	* CV* ^2^
True Value	NA	0.3	3.0	0.2	0.3	0.04	0.09	0.01
1-cohort	100	0.30 (4.4)	2.95 (9.5)	NA	NA	NA	NA	0.063 (573)
2 × 2-cohort	100	0.313 (13.8)	2.90 (12.0)	0.160 (57.2)	0.247 (35.6)	NA	NA	0.0177 (143)
3 × 3-cohort	100	0.304 (12.1)	2.95 (14.2)	0.161 (56.6)	0.250 (34.9)	NA	NA	0.0116 (168)
4 × 4-cohort	100	0.304 (12.4)	2.93 (12.2)	0.176 (55.3)	0.278 (44.2)	NA	NA	0.0116 (119)
5 × 5-cohort	100	0.301 (9.7)	2.97 (12.5)	0.178 (42.6)	0.283 (30.2)	NA	NA	0.00668 (84.8)
FOCE	19	0.314 (8.9)	2.94 (6.9)	NA	NA	0.0223 (62.6)	0.0530 (70.3)	0.0167 (146)
IMP	100	0.301 (65.9)	2.97 (66.1)	NA	NA	0.0276 (77.8)	0.0767 (77.1)	0.00359 (98.1)
SAEM	68	0.314 (10.6)	2.99 (9.6)	NA	NA	0.0496 (84.4)	0.0908 (52.3)	0.398E-15 (100)

*S.R.* Success rate

*NA* Not available

The success rates of all estimation methods for the dense datasets were 100%. For the sparse datasets all estimation methods but FOCE and SAEM were 100% successful. The success rate for FOCE was 19% and for SAEM 68%. Failures to provide parameter estimates with their standard errors were due to unsuccessful minimization of the objective function or a singular covariance matrix for parameter estimates.

The accuracy of estimates of * CL* and * V* for the dense dataset were less than 4% for all estimation methods. Their precision measured by %RRSME for FOCE, IMP, SAEM and 1-cohort methods was less than 7%, and for the remaining cohort methods less than 12%. The precision improved starting from the 2 × 2-cohort, down to the 5 × 5-cohort. The estimates of IIV parameters $\:{\omega\:}_{CL}$ and $\:{\omega\:}_{V}$ were more accurate for FOCE, IMP, and SAEM methods with less than 7% absolute difference from the true values whereas for the cohort methods this difference was less than 20% and decreasing with the cohort number. All methods underestimated the true values. The %RRMSE for FOCE, IMP, and SAEM methods was close to 35%. The 2 × 2-cohort method exhibited a similar precision, and it improved with the cohort number down to about 19% for the 5 × 5-cohort method. The estimates of residual variability $\:{CV}^{2}$were grossly overestimated by the cohort methods. The relative difference from the true value was 634% for 1-cohort and it decreased to 36% for the 5 × 5-cohort method. These differences were less than 6% for FOCE, IMP, and SAEM methods. The %RRMSEs for these methods were in the range 18% to 21%. For the cohort methods this range was 43% to 656%.

For the sparse data set the accuracy of estimates of * CL* and * V* were less than 5% for all estimation methods with the best relative difference from the true value less than 1% for the 5 × 5-cohort method. Their %RRSMEs for FOCE were less than 9%, for IMP about 66%, and for SAEM less than 11%. For the cohort methods %RRMSEs were less than 14%. The estimates of IIV parameters $\:{\omega\:}_{CL}$ and $\:{\omega\:}_{V}$ for FOCE and IMP methods were underestimated by 30% to 45% the true values and the SAEM method overestimated them by 24% and 1%, respectively. The cohort methods were more accurate with difference less than 20% and decreasing with the cohort number. All cohort methods underestimated the true values. The %RRMSE for FOCE, IMP, and SAEM methods were in the range 50% to 85% whereas for the cohort methods they were in the range 30% to 60%. The estimates of residual variability $\:{CV}^{2}$were inaccurate for all methods. The absolute relative difference from the true value for the cohort methods was in the range 16% to 529% and for FOCE, IMP, and SAEM methods in the range 64% to 100%. The mean estimate of $\:{CV}^{2}\:$by the SAEM was 0. The %RRMSE for all methods spanned the interval 84% to 573%.

To determine the impact of the number of cohorts on the model performance we analyzed the dense and sparse datasets for 5 cohort populations: 1-cohort (naïve data pool), 2 × 2-cohort, up to 5 × 5-cohort. For both datasets the accuracy of estimation of * CL*, * V*, $\:{\omega\:}_{CL}$, and $\:{\omega\:}_{V}$ did not change with increasing the cohort number. The cohort number influenced the precision of estimates of these parameters for the dense dataset. It did not impact the precision of estimates for the sparce dataset. The accuracy of estimation of $\:{CV}^{2}$ improved with the increasing of the cohort number for the dense dataset. It did so for the sparse dataset but only up to 4 × 4-cohort when it became 16% higher than the true value. For the 5 × 5-cohort the mean of $\:{CV}^{2}$ estimates was 33% lower than the true value. The precision of $\:{CV}^{2}$ estimates improved with the increasing cohort number for both datasets.

### Study II

**Fig. 3:**
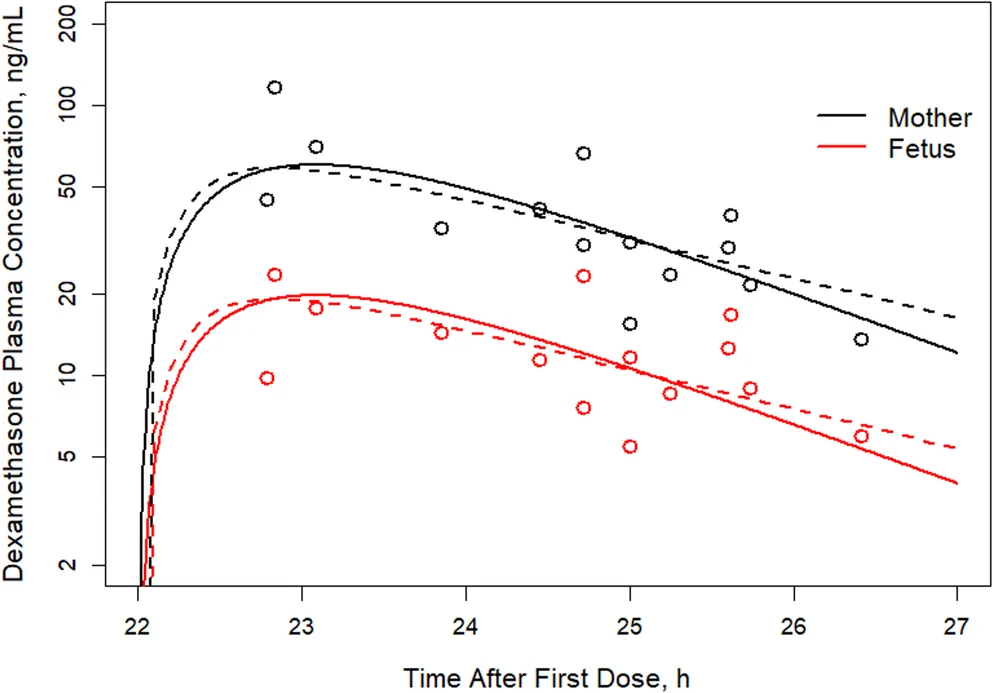
DEX plasma concentrations in maternal peripheral venous blood (black symbols) and fetal umbilical cord venous blood (red symbols) following intramuscular injections of 8 mg dexamethasone phosphate to 14 pregnant women prior to delivery by cesarean section. First dose was administered at 0 and second dose 22 h later. Lines represent population predictions of DEX plasma concentrations vs. time after first dose. Continuous lines represent 3-cohort model and dashed lines 1-cohort model (naïve pooled data)

The plots of typical $\:{C}_{m}$ vs. time and typical $\:{C}_{f}$ vs. time overlaid with observed data are shown in Fig. 3. The curves for mother and fetus are parallel due to assumed proportionality Eq. (24). There is a moderate improvement of fits between 1 and 3-cohort population models with the difference in the objective function value of 7.1. Parameter estimates are shown in Table 3. The estimates of typical values for * CL/F* and * FMR* are very similar for both models. The estimates of typical values for * V/F*, and $\:{k}_{a}$ differ between two models. However, the relative standard errors of these estimates are high for the 3-cohort population model that might result in no statistically significant difference. The over 100% values of %RSE for $\:{k}_{a}$ are a consequence of lack observations during the onset of plasma concentration time courses that are most informative about the absorption rate. The biggest difference between the two models is for the estimates of variances of residual variability $\:{{\sigma\:}_{m}}^{2}$ and $\:{{\sigma\:}_{f}}^{2}$ with 2.4-fold and 2.3-fold decreases, respectively. The estimate of the between cohort variability $\:\omega\:\:$ of $\:\mathrm{l}\mathrm{o}\mathrm{g}(CL/F)$ for the 3-cohort model resulted in the variance $\:{\omega\:}^{2}=0.09$.

**Table 3:** Parameter estimates and their relative errors (%RSE) for 1-cohort and 3-cohort population models

Parameter	Estimate (%RSE) *N* = 1	Estimate (%RSE) *N* = 3
CL/F, L/h	34.7 (18)	38.8 (21)
V/F, L	103 (23)	79.6 (59)
k_a_, 1/h	2.92 (127)	1.63 (147)
FMR	0.329 (7)	0.329 (7)
$\:\omega\:$	NA	0.30 (11)
$\:{{\sigma\:}_{m}}^{2}$	0.145 (30)	0.0611 (54)
$\:{{\sigma\:}_{f}}^{2}$	0.161 (28)	0.0695 (38)
OFV	-24.6	-31.7

*OFV* Objective function value

**Fig. 4:**
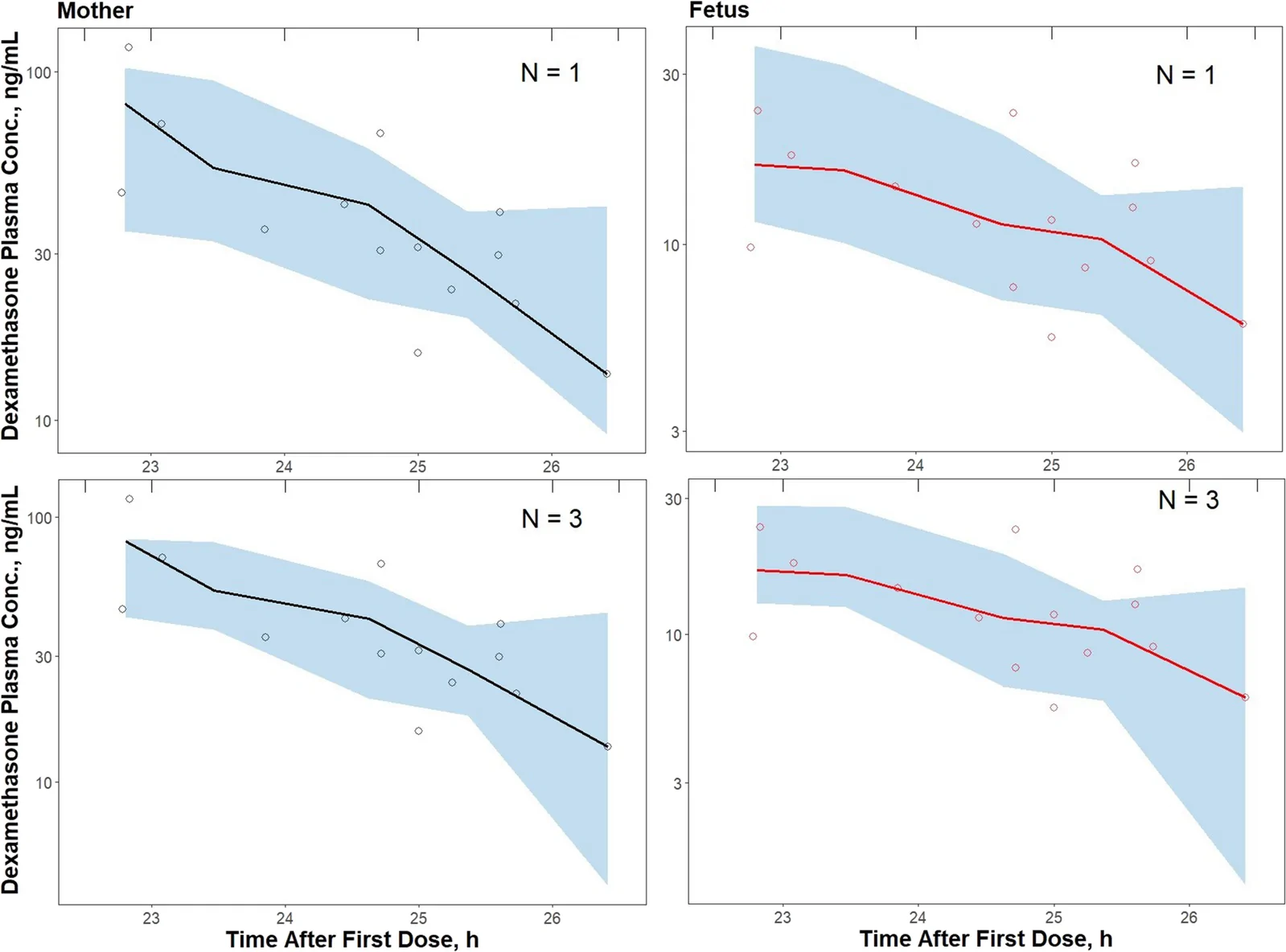
Visual predictive plots for 1 and 3-cohort population models of DEX in parturient women. Symbols are observed DEX plasma concentrations, and the lines are the median observations. The shaded areas are 95% prediction intervals for the median calculated from simulated 1000 populations. The 2.5 and 9.75 percentiles of observations could not be calculated because of sparse data

The diagnostic plots of observed vs. predicted (Fig S1) confirm good fits of the observed data by both models. The correlation coefficients for the log-transformed data were r^2^ = 0.73 (1-cohort) and r^2^ = 0.88 (3-cohort). The weighted residuals vs. time plots do not imply misspecification of the additive residual error models (Fig. S2). The vpc plots consist only of the median observed and 95% CI for the model predicted median (Fig. 4). The sparse data prevented calculation of meaningful 5th and 95th percentiles for the observations. The observed medians are inside the 95% CI bands for predictions. Also, the 95% CI bands for the 3-cohort model are narrower than the bands for the 1-cohort model (Fig. 4).

**Fig. 5:**
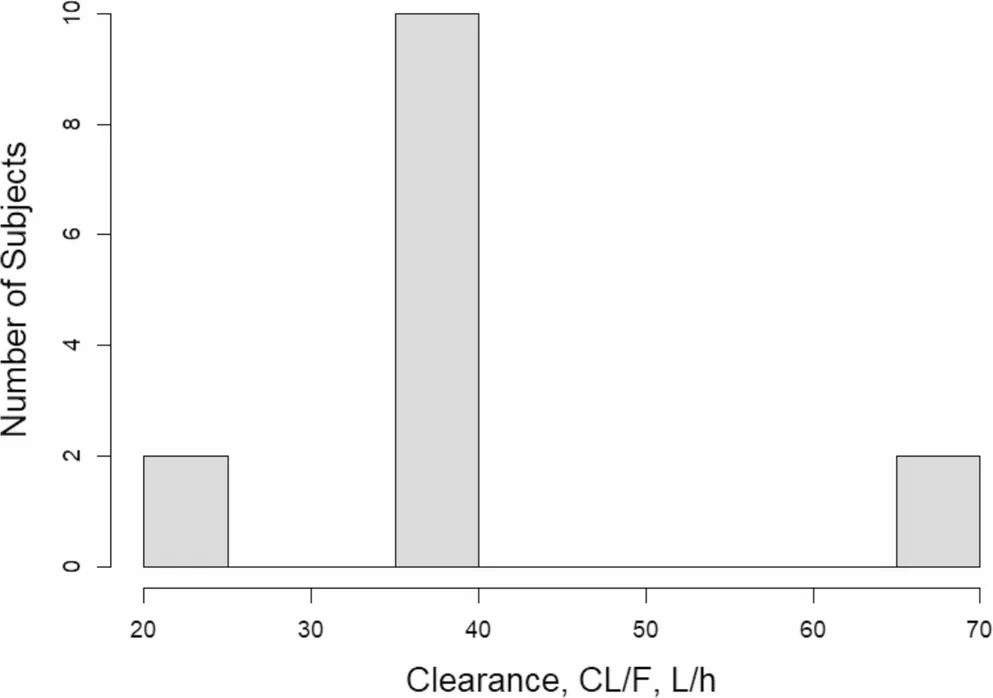
Frequency histogram of estimated individual apparent DEX clearances of parturient women. Three bins are representative of three cohort populations

The specific feature of a cohort population model is that each subject must belong to one of the cohorts. We used the probability of finding a subject in a subpopulation of the mixture population model as the basis for assigning individual * CL/F* values calculated from the nodes of the Gauss-Hermite quadrature. The 3 allowable values were 23.1, 38.8, and 65.2 L/h. The frequency histogram of * CL/F* distribution among subjects is shown in Fig. 5. Out of 14 subjects, 2 were in the lowest clearance cohort, 10 in the middle cohort, and 2 in the highest clearance cohort.

## Discussion

Our proof-of-concept study aimed to assess whether a cohort estimation method can outperform existing parameter estimation methods for nonlinear mixed effects models, such as FOCE, IMP, and SAEM where only sparse data are available. For comparison, we also simulated a dense dataset that contained sparse data. We simulated 100 datasets to evaluate success rate of the estimation method, as well as the accuracy and precision of parameter estimates. Additionally, we tested up to 5 cohort populations to investigate how the number of cohorts impacts the parameter estimates.

The cohort methods were robust both for dense and sparse datasets as their success rates of estimation were 100%. Only the IMP method had the100% success rates for both scenarios. The FOCE and SAEM method failed to estimate model parameters and calculate their standard errors for the sparse dataset more than 30%. The FOCE, IMP, and SAEM methods were more accurate and precise for estimation of * CL* and * V* for the sparse dataset than the multi-cohort methods but they were less accurate and precise in estimations of the IIV parameters $\:{\omega\:}_{CL}$ and $\:{\omega\:}_{V}$. All methods were inaccurate and imprecise, estimating the residual variability $\:{CV}^{2}$ for the sparse dataset. This is expected for data consisting of only one observation per subject where the calculation of the expected population means of model parameters is based on the individual estimates. In absence of individual estimates, an estimate of population mean is used. The multi-cohort method does not estimate between subject variability but rather between cohort variability. Consequently, the residual variability estimate is determined by the difference between the cohort mean and individual observations. In the presented study, the between cohort variability mimicked the true IIV in expense of the residual variability that was underestimated for the sparse data. More studies should be performed to quantify the trade between cohort variability and residual variability in estimation of IIV parameters by the cohort population method for more complex nonlinear mixed effect models.

The theoretical basis for using the Gauss-Hermite quadrature nodes and weights for construction of the cohort population is that it approximates the likelihood of observations with log-normally distributed * CL* and * V* when the cohort number increases to infinity [8]. This implies that for large enough cohort number the cohort method of estimation should not much differ from FOCE, IMP, or SAEM methods regardless of the data. We observed stark differences between these methods, particularly in estimating the residual variability, up to the 5 × 5-cohort which implies a higher number than 5 should be used to observe the resemblance. Our implementation of the cohort population model in NONMEM used mixture populations that required definitions of individual * CL* and * V* for each of N^2^ subpopulations. For N = 5 we reached 25 subpopulations that exceeded the number of 20 subjects in each dataset. However, even 4 × 4-cohort sufficed to estimate * CL*, * V*, $\:{\omega\:}_{CL}$, and $\:{\omega\:}_{V}$ with 13% accuracy and precision for the dense dataset.

To demonstrate that sparse data with only one observation per subject is a meaningful scenario, we referred to clinical studies with parturient women where data are limited only to blood samples drawn after childbirth. In particular, we selected a study aimed at quantification of DEX plasma concentrations in mothers and fetuses following antenatal IM administration of 8 mg dexamethasone-phosphate. The one compartment model was applied to fit maternal and fetal data with one observation per subject (mother and fetus). Because births occurred at random times after the first dose, a relatively wide time span was covered, ranging 0.8 to 4.4 h. This wide time interval included onset, peak and decline in maternal DEX plasma concentrations, allowing for identification of one-compartment model parameters.

The maternal plasma and fetal cord blood concentrations of DEX obtained from [12] are part of a larger data set used to develop a minimal physiologically based PK model [13]. The estimated value of * CL* reported in this study (with * F=1)* was 26.6 L/h which is similar to our estimate of typical value for * CL/F.* Tsuei et al. [12] reported * CL=33.5* L/h. The value of maternal blood volume at gestational age of 39 weeks was 5.7 L. Our estimates of * V/F* L are greater than the plasma volume but similar to values reported in [13]. The difference can be attributed to plasma protein binding (66.6% [12]), tissue binding, fractional bioavailability * F*, and use of 8 mg dose of dexamethasone phosphate rather than 6.67 mg of active DEX. Our estimates of the absorption rate constant $\:{k}_{a}$ is 1.63 h^−1^ and it is 1.8-fold bigger than the estimate of 0.89 h^−1^ in [13]. In the absence of absorption phase data in our observations, these estimates were obtained with high %RSEs and they should be interpreted with caution. The clinically important fetal to maternal plasma concentration ratio * FMR* was estimated with good confidence and its value agrees with 0.45 and 0.31 reported in [12] and [13], respectively.

We attempted to estimate IIV for all PK parameters using the 3-cohort method. The final model estimated IIV for * CL/F* while only 1-cohort estimates of * V/F*, $\:{k}_{a}$, and * MFR* were possible. Interestingly, the 3-cohort model performance was not very different from the 1-cohort model (naïve pooled data) using the diagnostic plots and %RSE for comparison. An improvement was observed comparing r^2^ and OFV values. This improvement can be attributed to partitioning data variability into between-cohorts and residual variabilities. The estimated 3-cohort * CL/F* values can be used for classification of subjects as slow, medium, and fast metabolizers since DEX is predominantly cleared by hepatic metabolism. The results of this study demonstrate robustness of the cohort estimation methods for populations with as extreme sparse data as one observation per subject.

Our goal was to introduce the concept of cohort estimation and compare it to the standard EM estimation methods. We did not attempt to compare the cohort estimation method with the non-parametric and Bayesian estimation methods that are applied to sparse data analysis. Further studies are necessary to assess pros and cons of these approaches.

In summary, we introduced an estimation technique for mixed effect models that stems from non-parametric methods of estimation and fully parametric mixture models with cohort population as a central concept. We showed that the cohort estimation can quantify the variability in (log)normal populations where between-cohort variability is a substitute for between-subject variability. The cohort estimates of IIV can be obtained for sparse data with better accuracy and precision than the standard maximum likelihood estimates. Finally, we showed that the cohort population estimates provide meaningful quantification of PK parameters for drugs studied in clinical trials where sparse data are inherent. Our results should be viewed as introductory and more thorough studies of properties of cohort population models are necessary to support our conclusions.

## Appendix 1

### NONMEM control stream and data set for 3-cohort DEX population model

$PROBLEM DEX GAUSS-HERMITE

$ABBR DERIV2=NO

$INPUT ID TIME DV MOTHER CP

$DATA DEX.csv IGNORE=C

$PRED

ksi1=-1.224744871391589*SQRT(2.0)

ksi2=0.0

ksi3=1.224744871391589*SQRT(2.0)

OM1=THETA(5)

IF(MIXNUM.EQ.1) CL = THETA(1)*EXP(ETA(1)+ksi1*OM1)

IF(MIXNUM.EQ.2) CL = THETA(1)*EXP(ETA(2)+ksi2*OM1)

IF(MIXNUM.EQ.3) CL = THETA(1)*EXP(ETA(3)+ksi3*OM1)

V= THETA(2)

KA=THETA(3)

FMR=THETA(4)

DOSE= 8000.0

T2=22.0 

KEL=CL/V

BESTSUB=MIXEST

A2=DOSE*KA/(KA-KEL)*(EXP(-KEL*T2)-EXP(-KA*T2))

IPRED=-10

IF (TIME.LT.T2) IPRED=LOG((DOSE/V)*KA/(KA-KEL)*(EXP(-KEL*TIME)-EXP(-KA*TIME)))

IF (TIME.GE.T2) IPRED=LOG(A2/V+(DOSE/V)*KA/(KA-KEL)*(EXP(-KEL*(TIME-T2))-EXP(-KA*(TIME-T2))))

Y = (IPRED*MOTHER+(LOG(FMR)+IPRED)*(1-MOTHER))+MOTHER*EPS(1) +(1-MOTHER)*EPS(2)

$MIX

NSPOP=3

PI=4*ATAN(1.0)

W1=0.2954089751509/SQRT(PI)

W2=1.181635900604/SQRT(PI)

W3=0.2954089751509/SQRT(PI)

P(1)=W1

P(2)=W2

P(3)=W3

$THETA

(0,37.6,) ; 1: CL L/h

(0,95, ) ; 2: V L

(0,3.9,) ; 3: KA 1/h

(0,0.334,) ; 4: FMR

(0,0.324,) ; 5: OM1

$OMEGA 

0.0 FIX 

0.0 FIX 

0.0 FIX 

$SIGMA

0.1

0.1

$EST METHOD=CONDITIONAL PRINT=1 SIG=3 MAXEVAL=9999 NOABORT

$COV PRINT=E

$TABLE ID TIME IPRED MOTHER CP BESTSUB NOPRINT ONEHEADERFILE= DEX.tab

### Data file DEX.csv

CID TIME DV MOTHER TSLD CP

1 22.78 3.807 1 0.7833 45.0

1 22.78 2.282 0 0.7833 9.8

2 22.83 4.762 1 0.8333 117.0

2 22.83 3.165 0 0.8333 23.7

3 23.08 4.257 1 1.083 70.6

3 23.08 2.879 0 1.083 17.8

4 23.85 3.564 1 1.850 35.3

4 23.85 2.674 0 1.850 14.5

5 24.45 3.726 1 2.450 41.5

5 24.45 2.434 0 2.450 11.4

6 24.72 3.421 1 2.717 30.6

6 24.72 2.028 0 2.717 7.6

7 24.72 4.199 1 2.717 66.6

7 24.72 3.148 0 2.717 23.3

8 25.00 3.444 1 3.00 31.3

8 25.00 2.460 0 3.00 11.7

9 25.00 2.747 1 3.00 15.6

9 25.00 1.705 0 3.00 5.5

10 25.25 3.165 1 3.25 23.7

10 25.25 2.152 0 3.25 8.6

11 25.60 3.391 1 3.60 29.7

11 25.60 2.542 0 3.60 12.7

12 25.62 3.674 1 3.617 39.4

12 25.62 2.827 0 3.617 16.9

13 25.73 3.073 1 3.733 21.6

13 25.73 2.197 0 3.733 9.0

14 26.42 2.610 1 4.417 13.6

14 26.42 1.792 0 4.417 6.0

## Supplementary Information

Supplementary Material 1 (DOCX 231 KB)

## Data Availability

Data is provided within the manuscript.
