# Supplementary material for: Cohort population analysis of sparse data: Dexamethasone pharmacokinetics in mother and fetus based on blood sampling at birth
Source: J Pharmacokinet Pharmacodyn. 2026 Jul 6;53(5):36. doi: 10.1007/s10928-026-10048-5 (PMC13337934; doi:10.1007/s10928-026-10048-5)
Supplement: Supplementary file 1 — Supplementary Material 1 (DOCX 231 KB) [file 10928_2026_10048_MOESM1_ESM.docx]

**Supplementary Material**

**Cohort Population Analysis of Sparse Data: Dexamethasone Pharmacokinetics in Mother and Fetus Based on Blood Sampling at Birth**

**Wojciech Krzyzanski**

**Table S1a.** Nodes for Gauss-Hermite quadrature rules for $2\leq N\leq5$ [10].

| $N$ | $\xi_{1}^{(N)}$ | $\xi_{2}^{(N)}$ | $\xi_{3}^{(N)}$ | $\xi_{4}^{(N)}$ | $\xi_{5}^{(N)}$ |
| --- | --- | --- | --- | --- | --- |
| $2$ | -0.70710  67811  86548 | 0.70710  67811  86548 |  |  |  |
| $3$ | -1.22474  48713  91589 | 0.00000  00000  00000 | 1.22474  48713  91589 |  |  |
| $4$ | -1.65068  01238  85785 | -0.52464  76232  75290 | 0.52464  76232  75290 | 1.65068  01238  85785 |  |
| $5$ | -2.02018  28704  56086 | -0.95857  24646  13819 | 0.00000  00000  00000 | 0.95857  24646  13819 | 2.02018  28704  56086 |

**Table S1b.** Weights for Gauss-Hermite quadrature rules for $2\leq N\leq5$ [10]

| $N$ | $w_{1}^{(N)}$ | $w_{2}^{(N)}$ | $w_{3}^{(N)}$ | $w_{4}^{(N)}$ | $w_{5}^{(N)}$ |
| --- | --- | --- | --- | --- | --- |
| $2$ | 0.886226  9254528 | 0.886226  9254528 |  |  |  |
| $3$ | 0.295408  9751509 | 1.18163  5900604 | 0.295408  9751509 |  |  |
| $4$ | 0.0813128  3544725 | 0.804914  0900055 | 0.804914  0900055 | 0.0813128  3544725 |  |
| $5$ | 0.0199532  4205905 | 0.393619  3231522 | 0.945308  7204829 | 0.393619  3231522 | 0.0199532  4205905 |

**Table S2.** DEX plasma concentrations at times after last dose in venous peripheral blood ($C_{m}$) and umbilical cord ($C_{f}$) of parturient women. Data were obtained from [12].

| Subject ID | Time after  last dose, h | $C_{m}$, ng/mL | $C_{f}$, ng/mL |
| --- | --- | --- | --- |
| 1 | 0.78 | 45 | 9.8 |
| 2 | 0.83 | 117 | 23.7 |
| 3 | 1.08 | 70.6 | 17.8 |
| 4 | 1.85 | 35.3 | 14.5 |
| 5 | 2.45 | 41.5 | 11.4 |
| 6 | 2.72 | 30.6 | 7.6 |
| 7 | 2.72 | 66.6 | 23.3 |
| 8 | 3 | 31.3 | 11.7 |
| 9 | 3 | 15.6 | 5.5 |
| 10 | 3.25 | 23.7 | 8.6 |
| 11 | 3.6 | 29.7 | 12.7 |
| 12 | 3.62 | 39.4 | 16.9 |
| 13 | 3.73 | 21.6 | 9 |
| 14 | 4.42 | 13.6 | 6 |

**Table S3.** The relative changes from the true values of model parameters estimates obtained by fitting 100 datasets of N =20 subjects with DENSE observations.

| Estimation  Method | $\%R\Delta\theta_{CL}$ | $\%R\Delta\theta_{V}$ | $\%R\Delta\omega_{CL}$ | $\%R\Delta\omega_{V}$ | $\%R\Delta\omega_{CL}^{2}$ | ${\%R\Delta\omega}_{V}^{2}$ | $\%R\Delta CV$^2^ |
| --- | --- | --- | --- | --- | --- | --- | --- |
| True Value | 0.3 | 3.0 | 0.2 | 0.3 | 0.04 | 0.09 | 0.01 |
| 1-cohort | 1.6 | 0.2 | NA | NA | NA | NA | 634 |
| 2×2-cohort | 3.4 | -1.7 | -17.6 | -19.8 | NA | NA | 196 |
| 3×3-cohort | 2.0 | -1.1 | -19.9 | -17.8 | NA | NA | 81.4 |
| 4×4-cohort | 2.3 | -0.7 | -2.2 | -3.5 | NA | NA | 53.8 |
| 5×5-cohort | 2.8 | -1.9 | -8.9 | -12.9 | NA | NA | 35.7 |
| FOCE | 1.7 | 3.4 | NA | NA | -5.5 | -6.4 | 5.8 |
| IMP | 0.9 | 0.3 | NA | NA | -6.1 | -6.1 | 3.1 |
| SAEM | 0.8 | 0.2 | NA | NA | -6.0 | -6.0 | 3.1 |

NA= not available

**Table S4.** The relative changes from the true values of model parameters estimates obtained by fitting 100 datasets of N =20 subjects with SPARSE observations.

| Estimation  Method | $\%R\Delta\theta_{CL}$ | $\%R\Delta\theta_{V}$ | $\%R\Delta\omega_{CL}$ | $\%R\Delta\omega_{V}$ | $\%R\Delta\omega_{CL}^{2}$ | ${\%R\Delta\omega}_{V}^{2}$ | $\%R\Delta CV$^2^ |
| --- | --- | --- | --- | --- | --- | --- | --- |
| True Value | 0.3 | 3.0 | 0.2 | 0.3 | 0.04 | 0.09 | 0.01 |
| 1-cohort | 2.2 | -1.7 | NA | NA | NA | NA | 529 |
| 2×2-cohort | 4.2 | -3.4 | -20.1 | -17.7 | NA | NA | 76.9 |
| 3×3-cohort | 1.3 | -1.8 | -19.4 | -16.6 | NA | NA | 15.6 |
| 4×4-cohort | 1.5 | -2.2 | -11.9 | -7.3 | NA | NA | 16.0 |
| 5×5-cohort | 0.3 | -0.9 | -11.2 | -5.7 | NA | NA | -33.3 |
| FOCE | 4.5 | -2.1 | NA | NA | -44.3 | -41.1 | 68.5 |
| IMP | 0.2 | -0.9 | NA | NA | -31.0 | -14.8 | -64.1 |
| SAEM | 4.5 | -0.2 | NA | NA | 24.1 | 0.9 | -100 |

NA= not available

**
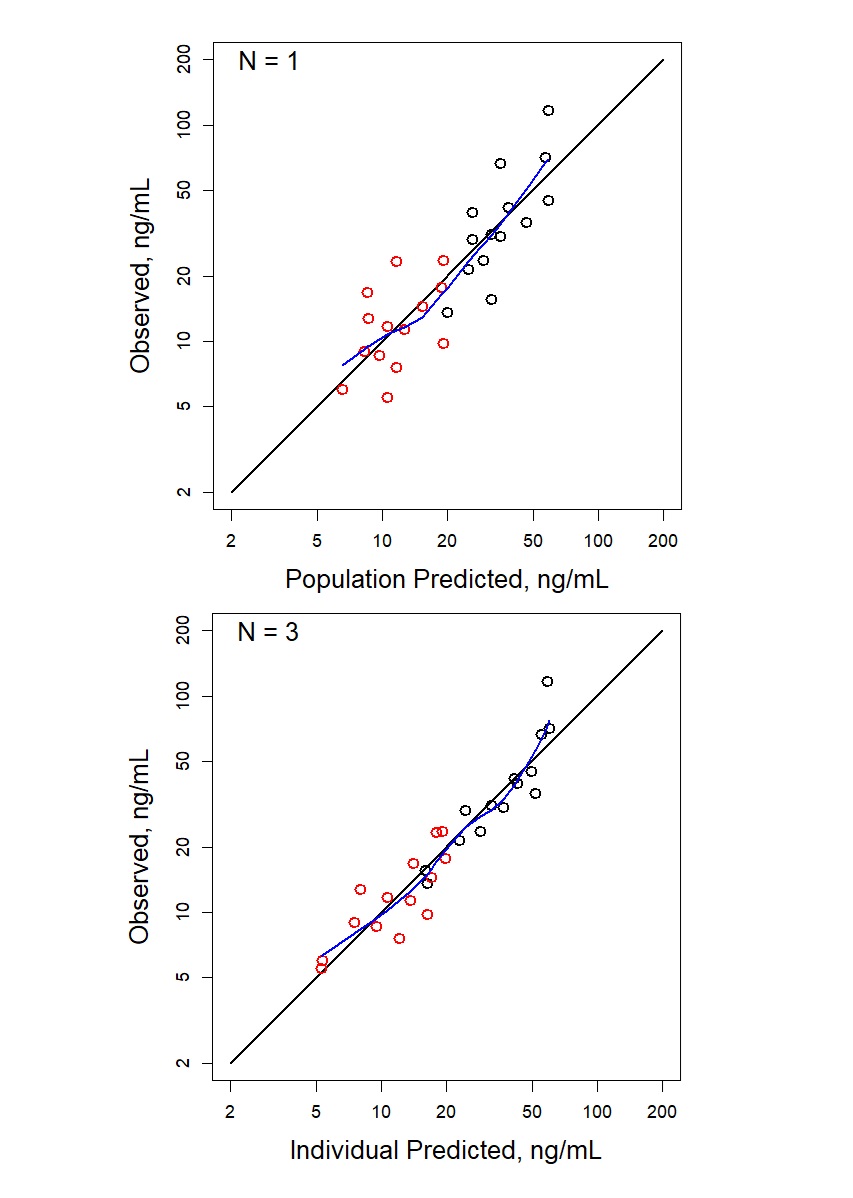
**

**Figure S1.** Observed vs. predicted diagnostic plots for the DEX population PK model. $N$ indicates the number of cohorts in the Gauss-Hermite distribution of $CL/F$. The black symbols represent maternal DEX plasma concentrations whereas the red symbols refer to the cord blood. The blue line is the LOESS curve.

**
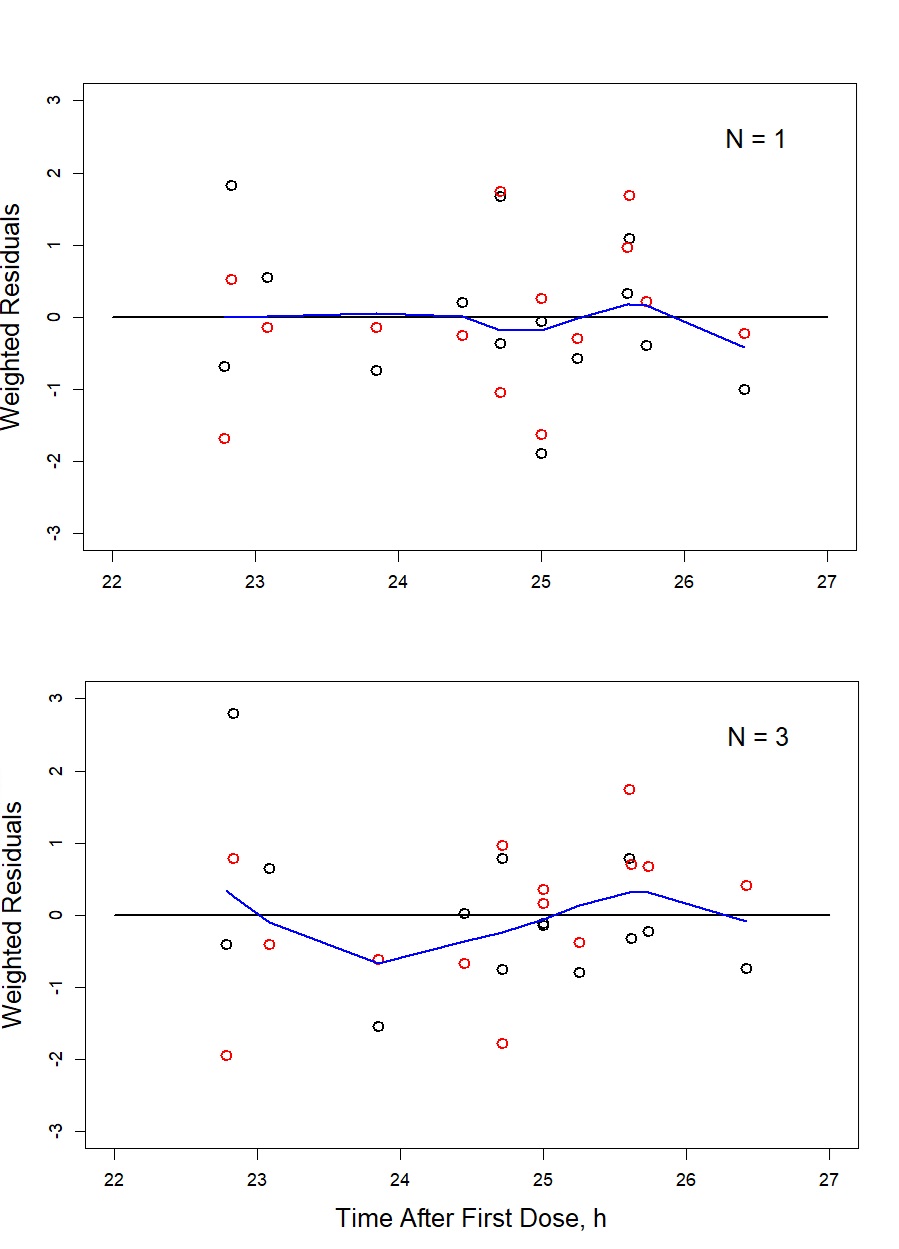
**

**Figure S2.** Weighted residuals vs. time diagnostic plots for the DEX population PK model. $N$ indicates the number of cohorts in the Gauss-Hermite distribution of $CL/F$. The black symbols represent maternal DEX plasma concentrations whereas the red symbols refer to the cord blood. The blue line is the LOESS curve.
